# Marine Antibody–Drug Conjugates: Design Strategies and Research Progress

**DOI:** 10.3390/md15010018

**Published:** 2017-01-13

**Authors:** Yu-Jie Wang, Yu-Yan Li, Xiao-Yu Liu, Xiao-Ling Lu, Xin Cao, Bing-Hua Jiao

**Affiliations:** 1 Department of Biochemistry and Molecular Biology, Second Military Medical University, Shanghai 200433, China; wyjcherish@163.com (Y.-J.W.); biolxy@163.com (X.-Y.L.); luxiaoling80@126.com (X.-L.L.); 2 Department of Medicinal Chemistry, China Pharmaceutical University, Nanjing 210009, China; yuyanli@cpu.edu.cn; 3 Shanghai Institute of Organic Chemistry, Chinese Academy of Sciences, Shanghai 200032, China

**Keywords:** marine toxins, antibody–drug conjugates, monoclonal antibody, targeted therapy

## Abstract

Antibody–drug conjugates (ADCs), constructed with monoclonal antibodies (mAbs), linkers, and natural cytotoxins, are innovative drugs developed for oncotherapy. Owing to the distinctive advantages of both chemotherapy drugs and antibody drugs, ADCs have obtained enormous success during the past several years. The development of highly specific antibodies, novel marine toxins’ applications, and innovative linker technologies all accelerate the rapid R&D of ADCs. Meanwhile, some challenges remain to be solved for future ADCs. For instance, varieties of site-specific conjugation have been proposed for solving the inhomogeneity of DARs (Drug Antibody Ratios). In this review, the usages of various natural toxins, especially marine cytotoxins, and the development strategies for ADCs in the past decade are summarized. Representative ADCs with marine cytotoxins in the pipeline are introduced and characterized with their new features, while perspective comments for future ADCs are proposed.

## 1. Introduction

While cancer is one of the most serious diseases threatening human life, traditional chemotherapy and common antibody tumor therapy offer low efficacy due to weak target selectivity and tumor-killing efficiency. As an innovative therapeutic choice, antibody–drug conjugates (ADCs) demonstrate distinctive advantages of both cytotoxin drugs and antibody drugs in cancer treatment. The marine cytotoxins, including auristatins and their derivatives (mainly MMAE and MMAF), take important position in ADCs payloads, and fully demonstrate their highly toxic merit under the accurate guidance of monoclonal antibodies.

**Figure 1:**
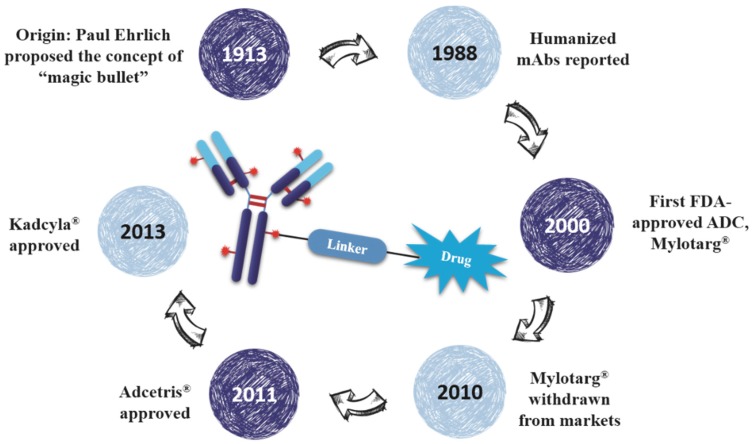
The brief history and schematic diagram of ADCs.

**Figure 2:**
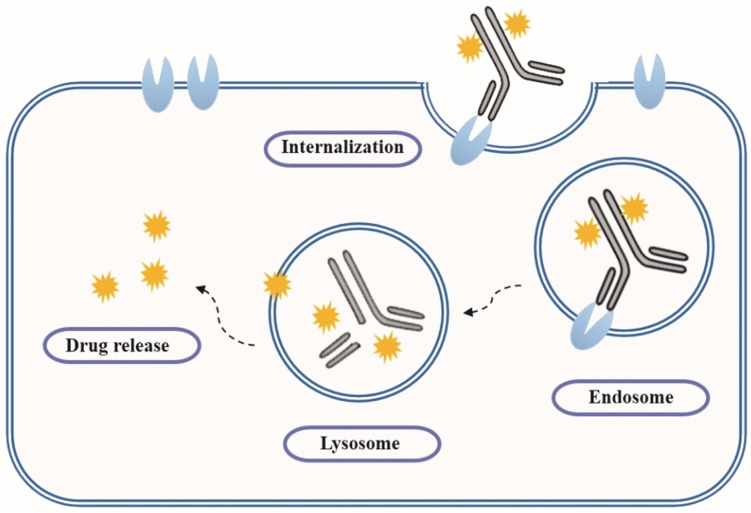
Action mechanism of ADCs.

As early as a century ago, German immunologist Paul Ehrlich proposed the concept of “magic bullet” which could selectively deliver cytotoxic drugs to tumor tissues by way of a targeting agent [1]. In the following decades, the evolved ADCs are well constructed with monoclonal antibody (mAb), highly cytotoxic compounds and a bioactive linker (Figure 1). The antibody efficiently carries cytotoxins to targets via specific antigen-antibody reactions, and the ADC-antigen complex is internalized by endocytosis [2]. Once internalized, the ADC moves into cancer cells and experiences further degradation to release drugs for killing tumor cells (Figure 2) [3]. Additionally, the conjugation strategy has an essential impact on the pharmacokinetics (PK) of cytotoxic drugs and can increase the half-life of these cytotoxins from hours to days [4].

**Figure 3:**
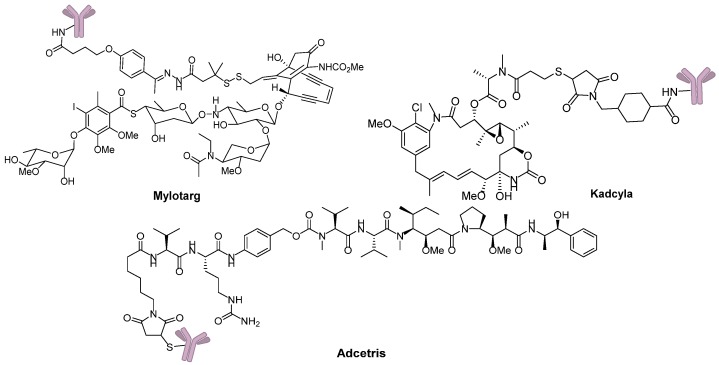
Three FDA-approved ADCs.

Eighty-seven years after Dr. Ehrlich’s proposal, the first ADC, Mylotarg^®^ (Pfizer, gemtuzumab ozogamicin), was approved for the treatment of acute myelocytic leukemia (AML) by the US Food and Drug Administration (FDA) in 2000. Though it was withdrawn from the market in 2010 for efficacy and overall survival problems [5], increasing attention and rapid progress towards ADCs have been made by pharmaceutical industry. In 2011, Seattle Genetics launched Adcetris^®^ (Brentuximab Vedotin), the second FDA-approved ADC for Hodgkin’s lymphoma (HL) and anaplastic large-cell lymphoma (ALCL) treatments [6]. Subsequently, Roche launched the third ADC drug Kadcyla^®^ (Trastuzumab Emtansine) towards HER2-positive breast cancers in 2013 (Figure 3) [7]. Till 2016, more than 50 ADCs’ clinical trial recruitments and enrollments have started all over the world. According to the prediction of “Antibody Drug Conjugates Market” (2nd Edition, published by Research & Markets), ~10 new ADCs will come into the clinical market in the next decade, and the overall ADCs market will reach 10 billion dollars by 2024.

## 2. ADCs in Clinical Trial

ADCs have grown to be one of the most promising tumor therapeutic drug research fields for their targeting, high efficiency, and low toxicity merits; the opportunity to develop brand new marine ADCs is coming. At present, there are more than 50 ADCs in the pipeline which have started their clinical trial recruitments or been in different clinical trial phases all over the world as shown in Table 1. Among these ADC candidates, Inotuzumab ozogamicin, an anti-CD22 calicheamicin conjugate developed by Pfizer, has started its Phase III study in treating relapsed and refractory acute lymphoblastic leukemia (ALL), and is expected to be launched in 2017. Around 20 ADCs are going to start Phase II recruitments or are in Phase II stage, and around 30 candidates have started their Phase I clinical trials [1,8]. Another exciting trend for ADC drugs is that the newer drugs have a wider range of indications. While early indication of ADCs mainly focused on leukemia and lymphoma, an increasing number of ADCs are now being aimed at solid tumors such as breast and ovarian cancers. For example, Glembatumomab vedotin, which is developed by Celldex, demonstrated high efficiency in Phase II study towards locally advanced or metastatic breast cancer—with an initial focus in triple negative disease; it is also in development for the treatment of Stage III and IV melanoma. Besides, Anti-PSMA ADC, RG7599, MLN0264 are recognized as efficient prostatic cancer, ovarian cancer and gastric cancer treatment agents.

## 3. ADCs Design

ADCs structurally consist of a monoclonal antibody, a bioactive linker, and payloads. For a successful ADC, four requirements should be simultaneously met: (1) suitable target; (2) highly specific antibody; (3) ideal linker; and (4) highly efficient cytotoxic drugs. ADC research involves many interdisciplinary subjects and high-tech research fields, which include the design and preparation of recombination mAbs, linker design, optimized conjugation of cytotoxic compounds, etc. At the same time, the drug:antibody ratios (DARs), antibody homogeneity, the distribution and metabolism of mAbs, effective release of drugs, and some other problems need to be taken into account. Finally, ADCs are obtained via the integration of all these high technologies. Any tiny problem will affect the safety and efficacy of ADCs [9,10].

### 3.1. Antigen and Indication

It is essential to choose a suitable target for designing a successful ADC, which is closely related with the efficacy and safety of drugs. An ideal biological target must overexpress on tumor cell membrane while only slightly express or does not express at all on normal cells [11,12]. ADCs are internalized after the antibody recognizes the antigen and cytotoxic drugs are released to kill the target cells. In the pipeline, most ADCs targets are tumor associated markers and proteins, such as EGFR, CD19, CD22, CD30, CD79a, etc. In accompany with more tumor associated markers identified, more novel antigens such as SLC44A4, PSMA, and 5T4 have been explored as new ADCs targets [1,13]. Moreover, as ADC targets, these tumor markers expressed on different kinds of tumor cells are also well recognized via biological identifications. These characterizations are extremely helpful to determine the potential hematologic and solid tumor indications of ADCs (Table 2).

Except for these traditional antigen targets, more and more novel targets are identified and have been applied to ADC design. CD70, a member of the neoplasm necrosis superfamily, is discovered to be highly expressed on the surface of different hematologic and solid malignancies but restricted to express in normal tissues. CD70 related ADC drug for immunological therapy was developed due to its rapid internalization when bound to the antibody [14]. SGN-CD70A, a CD70 targeted ADC, whose mAb is connected with pyrrolobenzodiazepines (PBDs) via a protease-sensitive linker, is in Phase I clinical trial for RCC and other lymphomas (NCT02216890). MDX-1203, developed by Medarex/BMS, takes the prodrug CC-1065 as payload and conjugates to the anti-CD70 mAb with a protease-sensitive linker [15]. Recently, MDX-1203 has completed Phase I in a multicenter, open-label, dose-escalation, multi-dose clinical study of subjects with advanced/recurrent ccRCC or relapsed/refractory B-cell NHL (NCT00944905).

PSMA (prostate-specific membrane antigen) is a type II inherent membrane protein specifically expressing on the prostate epithelial cell membrane. PSMA is an excellent target for ADCs with high selectivity that not only expresses on 90% of prostatic cancer but also 85% of neovascularization of solid tumors. The PSMA targeted mAbs could be rapidly internalized with receptor-mediated endocytosis after combining with the antibody [16,17]. The named anti-PSMA ADC whose mAbs connect with cytotoxins via a stable thioether linker has already finished phase II clinical trials in patients with metastatic castration-resistant prostate cancer (mCRPC) [18,19].

GPNMB (glycoprotein nonmetastatic melanoma protein b) is a type I transmembrane glycoprotein expressed in different tissues. Studies indicate that GPNMB up-regulates or abnormally expresses in several malignancies, including melanoma, breast cancer, and gliomas [20,21,22]. Glembatumumab vedotin, a fully human anti-GPNMB IgG_2_ antibody CR011 conjugating with MMAE via a protease-sensitive linker, is in phase I/II clinical trials for various metastatic melanoma and breast cancer [23,24]. Moreover, GPNMB is a potential target for other malignant neoplasms, including hepatocellular carcinoma and glioblastoma. Recently, phase II trial studies have shown its great potential in treating patients with osteosarcoma (NCT02487979).

Trophoblast glycoprotein (TPBG), also known as 5T4, is a carcinoembryonic antigen and a therapeutic target for several clinical anticancer drugs. Usually TPBG only expresses during embryonic development period while no expressions are detected in normal tissues [25]. However, the high expressions of 5T4 are detected from severe solid tumors, including ovarian, colorectal, and lung cancers [26,27,28]. A1-mc-MMAF, a humanized anti-5T4 IgG_1_ antibody conjugating to MMAF with a maleimidocaproyl linker, displays specific targeting efficacy for lung and breast cancer transplantation tumors; it is currently in clinical trial phase I [29].

### 3.2. Monoclonal Antibody

#### 3.2.1. Antibody Structure

The antibody is a glycoprotein belonging to the immunoprotein superfamily that can recognize the specific antigen. An antibody is constructed with two heavy chains and two light chains, which can be divided into constant regions and variable regions. The topological structure of the antibody can be seen as letter “Y”, while its variable regions situate on the terminals of two arms that can be regarded as the antigen-binding fragment (Fab). Moreover, a small fraction of amino acid residues changes intensively in the variable regions that decide the specificity of the antigen–antibody reaction, which is called the hyper-variable region. The glycosyl groups combine on the petiole of the “Y”, which is called the crystalline fragment (Fc).

According to different heavy chain types, antibodies could be classified as 5 isotypes, among which IgG is commonly used as the monoclonal antibody in clinical treatments for its long half-life character. IgG could also be split into four subtypes, IgG_1_, IgG_2_, IgG_3_, and IgG_4_, of which IgG_1_, IgG_2_, and IgG_4_ are more suitable for ADC design [30,31]. Additionally, since the monoclonal antibody has inherent anticancer properties, while IgG1 is able to generate stronger antibody-dependent cell-mediated cytotoxicity (ADCC) and complement-dependent cytotoxicity (CDC) towards cancer cells than IgG_2_ and IgG_4_, IgG_1_ is the most widely used IgG category in ADCs [32,33,34].

The antibody is a carrier for transporting drugs to target cells, and ADCs are mostly in the form of ADC-antigen complexes when internalizing into the cells. Some antibody–antigen complexes themselves have poor internalization character; however, their ADC conjugates can enhance their internalization process. For example, rituximab is an anti-CD20 antibody with poor internalization character, but its MMAE conjugates can improve their efficacy by optimizing the internalization of complexes [35].

#### 3.2.2. History of Therapeutic MAbs

The antibody plays an important role for ADCs in control of targeted elimination of cancer cells. With the rapid development of gene engineering, cell engineering, and antibody engineering, the monoclonal antibody has gradually undergone transformations from early murine and chimeric mAbs to humanized and fully human mAbs, from which the immunogenicity problems of the antibody are greatly reduced and their half-lives in blood are obviously prolonged. All these progress pave the way for improved efficacy of ADCs.

Three decades ago, murine mAbs were used in clinical research. However, compared with human IgG, the murine mAbs own short half-life, weakly binding to the human neonatal Fc receptor (FcRn) and low ADCC and CDC effects. They also produce allergic reactions and anti-drug antibodies (ADAs). As a result, they could not reach the satisfactory level of efficacy in cancer therapy [32,36]. Subsequently, through genetic engineering projects, chimeric mAbs were developed for overcoming the inherent immunogenicity and weak effector function of murine mAbs, by transplanting entire variable regions of a mouse antibody to the constant regions of a human antibody. Compared with the murine mAbs, the half-life of chimeric mAbs were prolonged, as well as immunogenicity were also reduced, however, the ADA problems were still remained [37,38]. More recently, only after the hyper-variable regions of murine were grafted onto the human antibody, and the humanized mAbs were generated, the ADA problems were basically solved as well as the mAb properties were greatly improved [39]. With the in vitro phage display technology and transgenic mice expressed human variable regions, full human mAbs with humanized variables and constant regions are developed. These humanized mAbs owns little immunogenicity and are more similar characters with human endogenous IgGs [40,41,42,43]. Nowadays, humanized and fully human mAbs have been the mainstream antibody applied in ADCs (Table 3) [34,44].

The high affinity between antibody and antigen is crucial for effective target killing. Studies have shown that Kd = 10 nM is the basic requirement for antibodies, since the antibodies can effectively accumulate on tumor sites when the value of Kd reaches 10^−7^ M [45]. In addition, the antibody should be stable and own a long half-life in blood, and could effectively trigger endocytosis. There are 125 antibodies which could recognize 89 specific antigens in human body; among these, 55 targeting antigens are related to anticancer diseases [34], while panitumumab and cetuximab are the tumor-targeting mAbs for targeting epidermal growth factor receptor (EGFR) [34,46]. Adcetris adopts the chimeric IgG_1_ (cAC10) that can recognize the CD30 receptor on the cells of Hodgkin’s lymphoma and anaplastic large cell lymphoma [47].

#### 3.2.3. Novel MAbs

Due to its antineoplastic activity of inherent ADCC and CDC, IgG_1_ is commonly used in ADCs; however, more improved IgGs have been proposed for new generation of ADCs. It has been found that the glycosylations play significant roles in the function and stability of antibodies. The interaction between lgG-Fc and receptor depends on the bilateral saccharide structures, which can affect the ADCC activity [48]. For example, Obinutuzumab (Gazyva) greatly improved its ADCC effect through glycoengineering modification, which supplies additional targeted killing towards cancer cells [49].

Novel bispecific antibodies have appeared. As specified, the binding domains of these antibodies own different functions simultaneously, for example one antibody can interact with two different types of target antigens [50]. Catumaxomab (Removab), the first bispecific mAb coming into the market in 2009, is considered as a trifunctional macromolecule, which can recognize CD3 on T cells, recognize EpCAM on other tumor cells, and facilitate ADCC for tumor cell killing [51].

**Figure 4:**
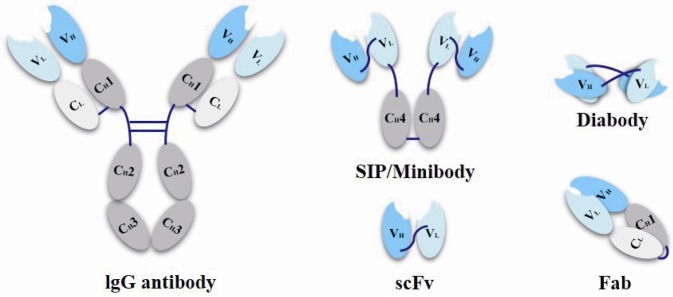
Schematic representation of miniaturization antibodies.

Antibody fragments as ADC carriers have also making striking progress. For traditional ADC drugs, only a few candidates are found aiming at solid tumor indications. It is mainly because that the large molecular weight of the full size antibody (~150 kDa) makes it difficult to penetrate capillary endothelial cell and traverse tumor’s extracellular space to interior part of solid tumor, as a result that only a small percentage of antibody can reach the targeting cells. Except for penetration and the inefficient extravasation of antibodies, “antigen barrier effect” is another problem, caused by their trapping by antigen on perivascular tumor cells [52,53]. To solve these problems, antibody fragments are taken into account [54]. Scientists prepared small molecular weight conjugates, such as single-chain variable fragments (scFvs, 25 kDa), antigen-binding fragment (Fab, 50 kDa), diabody (55 kDa), SIP/minibody (80 kDa) (Figure 4) with improved penetrability and increasing amount of drugs that could reach deeper tumor cells [45]. Some researches have found that the SIPs/miniantibodies exhibit the best results on the efficient tumor uptake and a suitable rapid plasma clearance when compared with other antibody fragments [55,56,57]. However, these smaller proteins also have their own problems with less half-life and rapid plasma clearance, so the higher binding affinity is even more important should be noted for utilizing these miniaturization antibodies, because the in vivo half-life as well as the drug efficacy will also be affected corresponding to the reduced molecular weights of these antibody fragments [58].

### 3.3. Site of Conjugation

#### 3.3.1. Non-Specific Conjugation

The conjugation site is the binding site between the monoclonal antibody and the linker-payload. Most ADCs in the pipeline adopt the traditional “non-specific conjugation” techniques, using the lysine and cysteine as ADCs conjugation sites. Firstly, the naked ε-amino group of lysine on the surface of the monoclonal antibody own excellent chemical reactivity and water solubility for chemical conjugations. The lysine sites can be directly conjugated under mild condensation conditions with linkers without processing or modification. Secondly, the thiol group of cysteine on the surface of mAb is also ideal conjugation site. However, the thiol groups of cysteines always exist in a disulfide bond form, including both intra-chain and inter-chain disulfide bonds. Commonly, the thiols used to connect linkers are obtained by inter-chain disulfide bond reduction, without affecting the conformation folding of the recognition sites [59,60]. Compared with the lysine conjugation sites, the superiority of cysteine sites is they own much lower heterogeneity, however, the disadvantage of cysteine sites is that their reduced disulfide bonds may affect the structure stability of entire ADCs.

#### 3.3.2. Site-Specific Conjugation

Usually an average of four to six toxin molecules could kill a target cell [61]. However, studies have indicated that the drug:antibody ratios (DARs) for these non-specific conjugation ADCs are probably distributed from one to eight, as a result the conjugated ADCs are a mixture of antibodies with different number of drugs. For example, the number of cytotoxic drug MMAE in Adcetris is between zero and eight with an average DAR value of 3.5 [62]. Recent researches showed a certain intracellular concentration of drugs determines the antitumor activity of ADC in vivo [63], so the higher DARs may be beneficial to deliver more drug to tumors. The inhomogeneity of the preparation of ADCs is a challenge for both drug production and quality control, and simultaneously has a great influence on in vivo distribution, metabolism, and efficacy. This would be the most important disadvantage for non-specific conjugations. In order to solve the inhomogeneity problem, scientists have proposed various site-specific conjugation strategies, for making unified ADC DARs. Commonly, the specific sequence of mAbs can be site-directed, modified, and introduced active functional groups as a chemical reaction site [64,65,66]. Extra cysteine or unnatural amino acids can be site-directed via these gene engineering methods; for example, serine and *p*-acetylphenylalanine (pAcPhe) could be used to replace cysteine through site-directed mutagenesis. Moreover, Bertozzi’s team utilized the special formylglycine-generating enzyme (FGE) to recognize the CXPXR sequence, by which they could realize the substitution of the cysteine with formylglycine quantitatively and site-specifically. Consequently, the introduced orthometric active formyl groups in the mAb can be the specific chemical reaction site to solve the inhomogeneity of ADCs [66].

**Figure 5:**
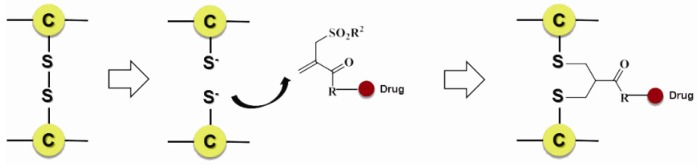
Schematic ThioBridge™.

**Figure 6:**
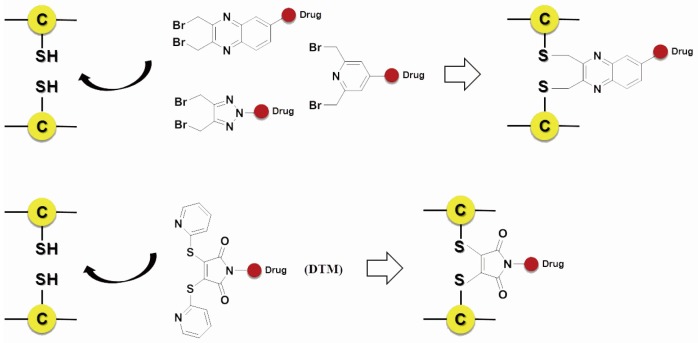
Schematic c-Lock™/K-Lock™.

Scientists also developed many other special organic chemical strategies for optimizing and increasing the coupling selectivity. For example, PolyTherics utilizes the characteristic of moderate distance of the two sulfydryls from the reduction of the disulfide bond in antibody. A special ThioBridge™ is formed via two-ring formation of Michael addition reactions, which has higher stability and better reaction specificity compared with traditional links. The single ingredient with four MMAE as the payload of the ADC prepared by ThioBridge™ technology accounts for 78% of all components, which is much higher than traditional preparation (Figure 5) [61,62,63,64,65,66,67,68]. Analogously, Sorrento and Concortis utilize cyclization via c-Lock™/K-Lock™ technology. This approach can obtain ADC drug almost without the use of naked antibodies, and its sulfur bridge bond is stable and has good reaction specificity. Igenica also adopts dithiopyridylmaleimide (DTM) to insert a dicarbon bridge (Figure 6).

**Figure 7:**
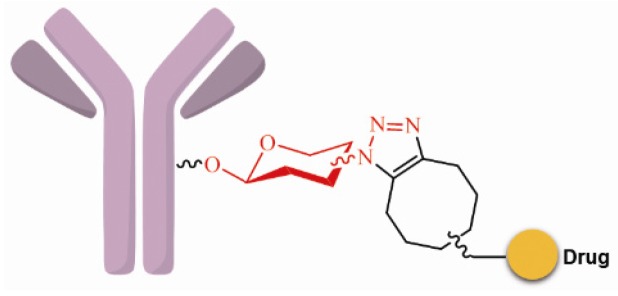
Schematic GlycoConnect™.

SynAffix initiated and came up with GlycoConnect™ that sugar units with azide, sulfydryl or chlorine atom could be introduced into the glycosyl areas of the antibody via recombination. These functional groups can be used specifically for coupling with the effector molecules; for example, the acetylenic bond can react with azide via “click chemistry”, and homogeneous and site-specific ADCs are obtained (Figure 7) [69].

**Figure 8:**
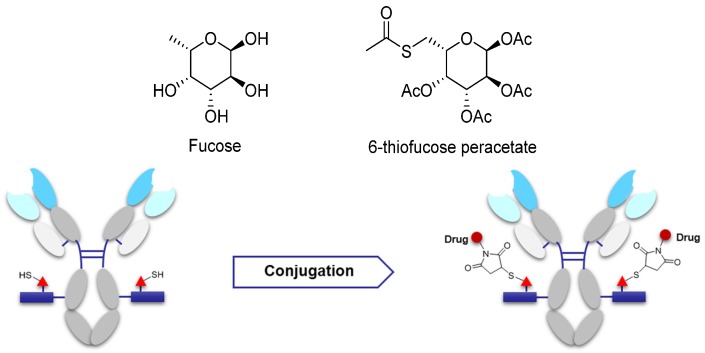
Schematic of antibody saccharides for conjugation and structures of fucose. Monosaccharide symbols used are as follows: triangle, fucose; rectangle, GlcNAc and mannose.

Some studies have suggested that saccharides play an important role in antibody function and pharmacokinetics. Non-natural saccharides can be integrated to the sugar chains of antibody via the metabolism to introduce novel structures with better activity or provide potential new sites for drug conjugation [70]. The non-natural saccharides connect with the linker-payload to obtain ADCs with high homogeneity via maleimide chemistry. The terminal fucose on the heavy chain N-linked saccharides of the antibody can be replaced with fucose analogs by fucosyltransferases VIII, and the saccharides with novel structures are obtained [71,72]. 6-Thiofucose peracetate is a fucose analog that offers a convenient site for conjugation (Figure 8); ADCs with this connection site have favorable activity, immunological specificity, and stability.

### 3.4. Linker

**Figure 9:**
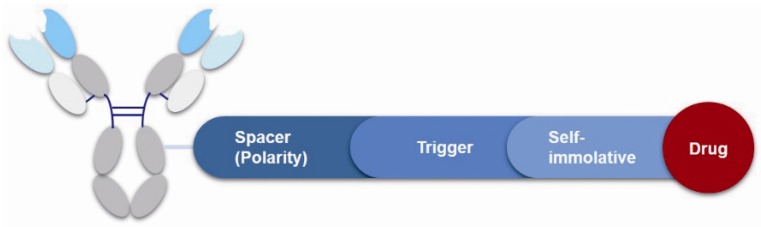
Structure of linker in ADCs.

The linker is the bridge to connect the antibody and drugs, which can be divided into the spacer, trigger, and self-immolative part (Figure 9). However, not all linkers contain all these parts. The linker decides the release of drugs in the cells and has a significant effect on the entire kinetic property of the drugs, their efficacies and so on. First, an ideal linker plays the role of connector and carrier, and guarantees that the ADC is soluble and stable in the aqueous solution. Second, the linker should keep considerably stable in the blood circulation to reduce the potential toxicity and side effects brought about from the early release of the drugs [73]. The earliest launched ADC drug Mylotarg only showed a narrow therapeutic index result from the instability of the hydrazone linker in plasma. Finally, when the ADC is internalized into the cell after antibody–antigen binding, the drug should be rapidly released under the action of lysosomes or other enzymes to kill the target cells.

#### 3.4.1. Spacer

**Figure 10:**
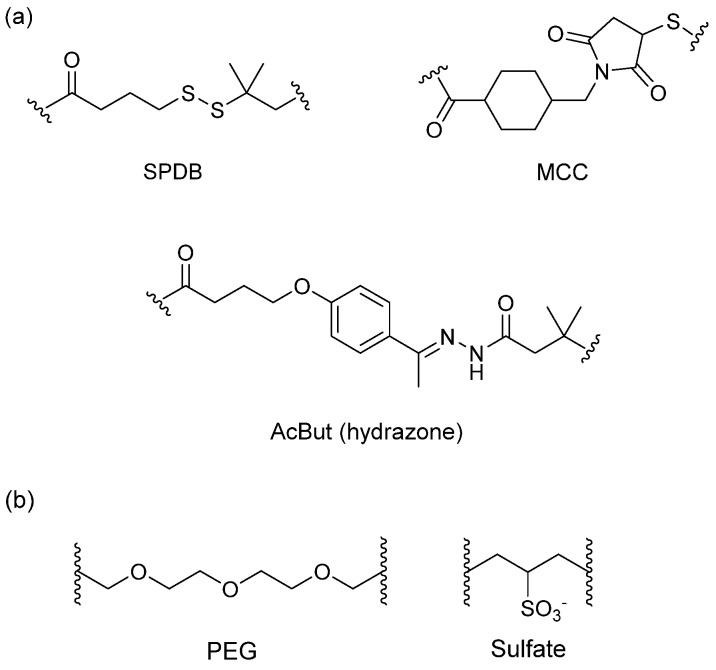
Structures of the spacer and polarity parts: (**a**) three representative connections of the linker to the antibody; and (**b**) the polarity of the linker.

The spacer is the part of the linker that connects to the monoclonal antibody. The linker with lysine as the connection site reacts with the ε-amino of lysine by acylation, mainly including lipophilic SPDB disulfide without charges, maleimidomethyl cyclohexane-1-carboxylate (MCC), sulfo-SPDB with a charged polar group, and the 4-(4-acetylphenoxy)butanoic acid (AcBut) spacer in the hydrazone linker (Figure 10a) [74,75,76]. Mylotarg, the first launched ADC, has an AcBut spacer, while Kadcyla adopted MCC. The maleimide, which has an excellent reactivity with sulfydryl, is usually introduced to connect to the cysteine in the antibody, including maleimidocaproyl (MC) and maleimidomethyl cyclohexane-1-carboxylate (MCC). Importantly, all known ADCs with the auristatin payload adopt MC to connect with the antibody, because the MC can provide enough space for proteases to recognize the valine-citrulline. However, maleimide is unstable in blood and may lead to early breaking of the drug-linker. In order to achieve a better level of efficacy, some studies have indicated that adding an amino group in the adjacent position of maleimide could effectively prolong maleimide’s half-life under the neutral plasma condition [77].

The overall polarity of ADCs also has important impact on their pharmacokinetics, efficacy, and DAR of ADC drugs. Usually, the drug loading capacity will be increased as much as possible to enhance the efficacy, however, most cytotoxins are hydrophobic, which causes aggregation and insolubilization when connecting to the antibody [78]. This limits the drugs loading to the antibody and decreases their affinity with antigens. Additionally, the hydrophobicity can generate multi-drug resistance since the P-glycolprotein will excrete these drugs out of tumor cells and cause reduced efficacy [79]. Consequently, a polarity part, such as a polyethylene glycol (PEG) or a sulfate group, can be added to the spacer to increase the overall hydrophilia and polarity of ADCs (Figure 10b) [80].

#### 3.4.2. Trigger

According to different action modes, the linker can be divided into cleavable and non-cleavable linkers as shown in Table 4. Due to different working mechanisms, a more suitable connection type can be chosen based on different target environments, payloads, and indications.

Specifically, the cleavable linker mainly includes three working modes. The first category is acid-sensitive linker, which releases drugs according to the changed environmental pH value. With the gradually decreasing of pH value from extracellular (pH = 7.4) to intracellular (pH = 5–6) and then to the lysosome (pH = 4.8), the acid-sensitive trigger is activated and the drugs released [81]. The hydrazone group belongs to the acid-sensitive linker, which has been applied in CMC-544 and IMMU-110. The second category is glutathione-sensitive linker, containing a disulfide bond that can be reduced by glutathione and release drugs taking the advantage of much higher intracellular glutathione concentration than that of plasma [74]. SAR3419 and IMGN901 are representative drugs of this category in the pipeline. The last category is protease-sensitive linker that recognizes and cleaves the specific peptide to release drugs by some proteases in the lysosome of tumor cells. Valine-citrulline (v-c) is the most common peptide used in current clinical research [82]. Adcetris, approved by the FDA, connects MMAE with the antibody by this linker [83,84]. Phenylalanine-lysine (p-l) and valine-alanine (v-a) have also been adopted in some other ADCs, such as Labetuzumab-SN-38 and SGN-CD70A. Addtionally, the β-glucuronide linker with a drug release mechanism based on β-glucuronidase is a recently developed useful linker, which indicated high efficacy at well-tolerated ADC doses [85].

A non-cleavable linker relies on the degradation of whole antibody after internalization of ADCs. The drugs are commonly released in the form of amino acid modification, while the antibody is completely hydrolyzed to amino acids in the lysosome. MCC and thioether are the most common non-cleavable linkers; for example, Kadcyla adoptes MCC to connect maytansinol with trastuzumab [75]. Bystander effects, another important consideration in ADC design, is a potential mechanism that the released payload is able to diffuse out of the cell and kill neighboring cells. Compared to cleavable linkers, non-cleavable linkers are even more stable in blood and not able to diffuse to adjacent cells. For instance, Kadcyla (T-DM1) releases lysine-DM1 with less membrane permeability instead of DM1, is not able to diffuse into neighboring cells [86].

**Figure 11:**
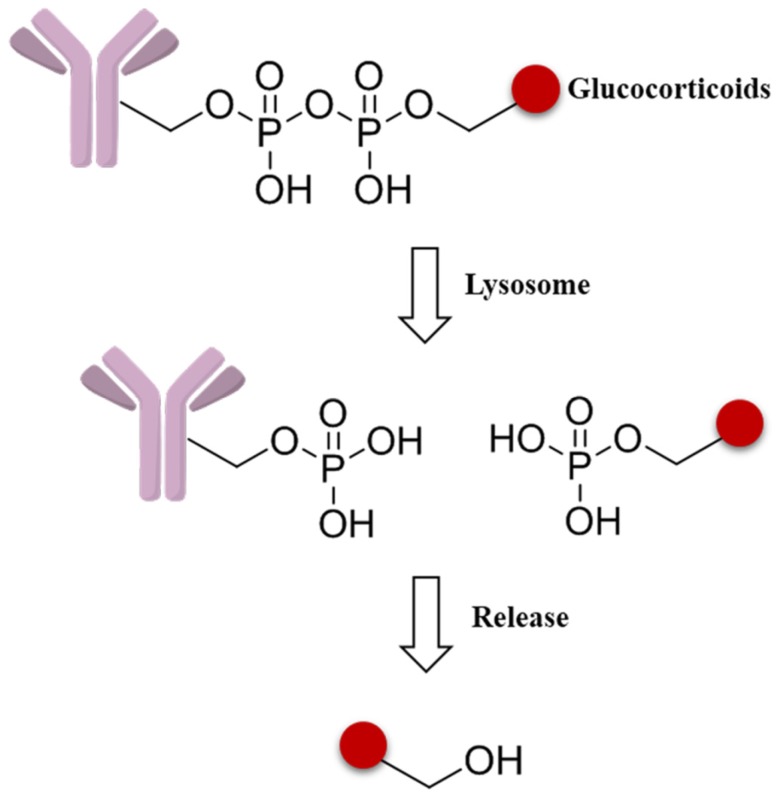
Schematic mechanism of a pyrophosphate ester linker.

As new effort to expand the applicability of ADCs beyond oncology, a novel pyrophosphate ester linker has been initiated to obtain the targeted delivery of glucocorticoids to immune cells (Figure 11). These phosphate linker–glucocorticoid molecules have high solubility in water, stability in blood, and a range of reactivities in the lysosome environment, and all these superior characters lead to wide applications in ADCs fields [87].

#### 3.4.3. Self-Immolative Spacer

**Figure 12:**
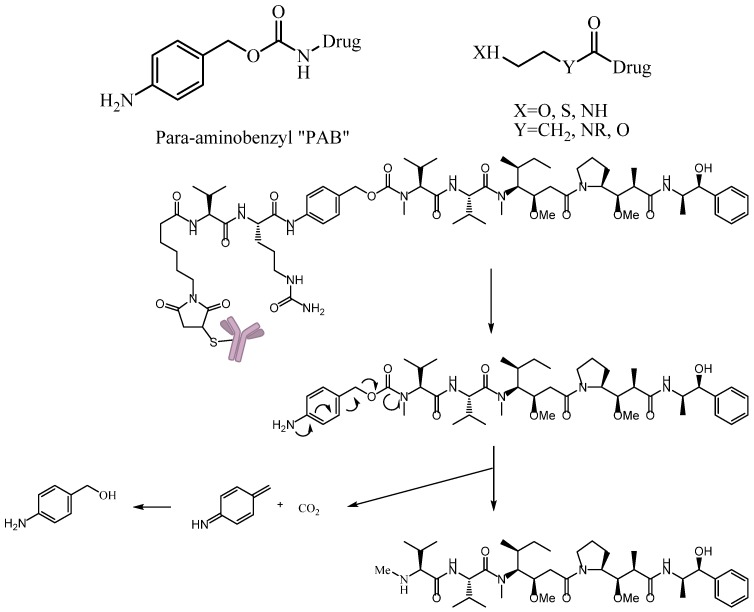
Schematic 1,6-elimination reaction of the self-immolative spacer after cleavage of the linker.

**Figure 13:**
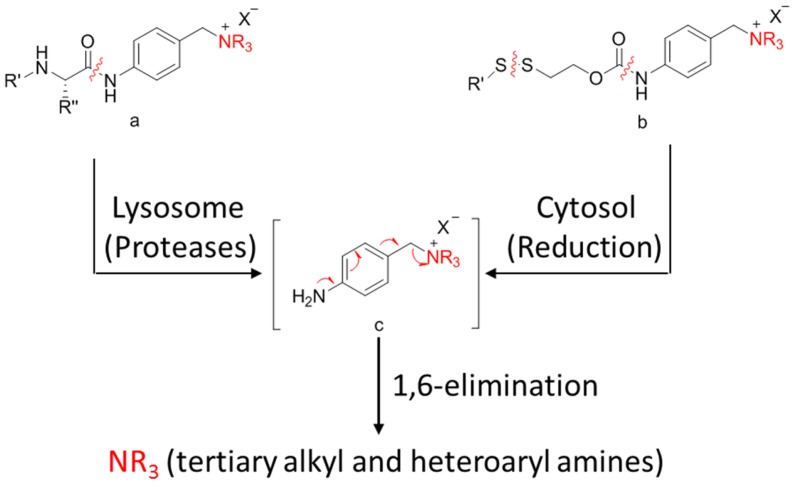
Schematic a bioreversible linkage based on a quaternary ammonium.

A self-immolative spacer is the part that still connects to the drug after the trigger activated and ADC cleavaged. It can release drugs through rapidly spontaneous intramolecular reactions, which is equivalent to the function of a prodrug [88]. Para-aminobenzyl (PAB) and heteroatoms are two main structures [89,90]. PAB is commonly connected to the peptide linker as the self-immolative spacer that can release active drugs by a 1,6-elimination reaction after the cleavage of the linker (Figure 12). Interestingly, Staben et al. described a bioreversible linkage based on a quaternary ammonium that can be used to connect various tertiary and heteroaryl amines to the antibody. It shows common cleavable ADCs with this new connection are effective and stable in vitro and in vivo (Figure 13). Besides, studies with a tertiary-amine-containing antibody–antibiotic conjugate show that the appropriate stability and release characteristics brought an unexpected improvement in activity over the carbamate [91].

### 3.5. Cytotoxic Payload

**Figure 14:**
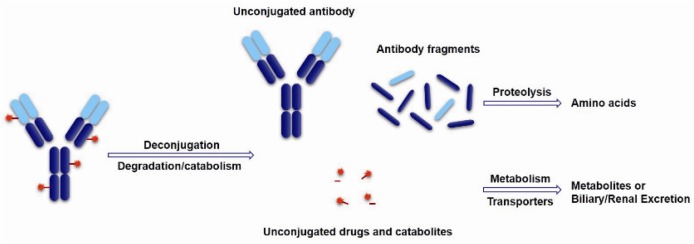
Schematic of theoretical ADCs metabolism pathways.

The cytotoxic drugs, which acting as the payload of ADCs, determine the therapeutic effects of ADC drugs. ADCs belong to proteinic drugs, which can be disaggregated into unconjugated antibodies and toxins or directly disassimilated into some antibody fragments and decomposition products of the drugs during the targeting therapy. Subsequently, the unconjugated antibody and antibody fragments further turn into amino acids under proteolysis. These toxins or drug catabolites can metabolize via cytochrome or non-cytochrome enzymes or are transported by P-gycoprotein and excreted via the biliary or renal pathway (Figure 14). Generally, only 1%–2% of the injected dose of ADCs can effectively reach the intended tumor target and come into action, which are largely different from small molecule drugs for their unique biodistribution and metabolism characters [92]. Therefore, the cytotoxinic drugs usually need very high efficiency and sensitively killing effects on target cells, whose IC_50_ should be up to 0.01–1 nM. Moreover, the payloads need to be stable in blood circulation and lysosomes. A relatively small molecular weight, a long half-life, and weak immunogenicity are also necessary characters for the toxins. For another, Li et al. studied the bystander effects of two auristatin payloads, MMAE and MMAF, which have similar structures. However, MMAF is more hydrophilic and less membrane permeable than MMAE. The result shows MMAF has a higher cytotoxinic activity than MMAE, consistent with its lower membrane permeability. All of this indicates that membrane permeability and the biophysical properties of the released payload are required for bystander effects [63]. It is particularly important to select appropriate payloads except for linker selection.

Currently, there are two main working mechanisms for ADC payloads: (1) disturbing the mitosis process by binding to microtubule targets; and (2) disrupting the DNA duplication process via splitting decomposition and alkylation targets. Some clinical chemotherapeutic drugs including vinca alkaloids, paclitaxel, amethopterin, and doxorubicin, were used as ADC payloads in early stage. However, most of these trials were failed attributed to their low curative effect, heterogeneity, and lacking of specific targets [5].

**Figure 15:**
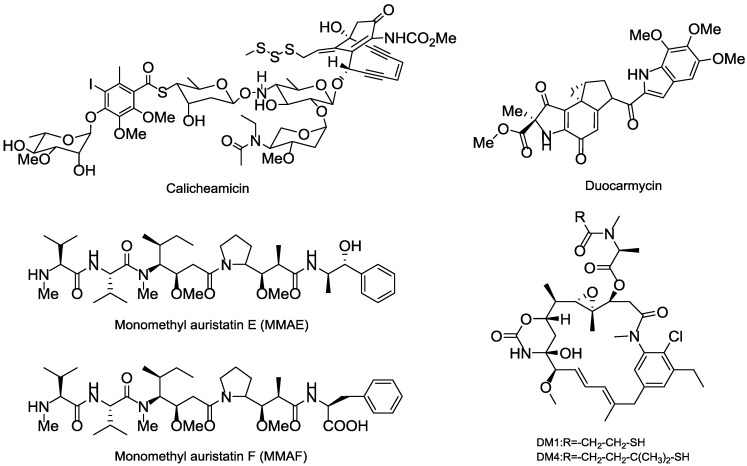
Several representative cytotoxinic drugs used as payloads in ADCs.

Thus far, all ADCs in the market or used in clinical trial research have adopted the highly toxic natural product or their derivatives as the payloads, of which four toxins, including calicheamicins, duocarmycins, auristatins and maytansines, are heavily relied. The calicheamicins and duocarmycins are DNA damage agents, while the auristatins and maytansines are tubulin inhibitors. Among that, Auristatins applied in ADCs mainly include monomethyl auristatin E (MMAE) and monomethyl auristatin F (MMAF), and the maytansines commonly used as payloads are DM1 and DM4 (Figure 15) [93,94].

#### 3.5.1. Terrestrial Payloads

Calicheamicin is a semisynthetic natural product separated from the soil microorganism *M. echinospora calichensis* that shows subpicomolar grade potency. The aryltetrasaccharide structure in calicheamicins can tightly bind to the minor groove of DNA that eventually results in DNA double-strand break and cell death [95,96]. *N*-acetyl-γ-calicheamicin is the derivative of calicheamicin that was used as the payload of the approved gemtuzumab ozogamicin (Mylotarg); however, Mylotarg was later withdrawn for unsatisfactory efficacy and safety reasons [97].

Duocarmycin is a derivative of the natural product isolated from the bacteria *Streptomyces* sp. that can bind to the AT-rich regions at DNA’s minor groove and induce irreversible alkylation to disrupt the DNA and cause cell death [98]. A novel prodrug analog of duocarmycin as the payload was connected to a fully human mAb CD70 to get the MDX-1203 that is now in phase I (NCT00944905) [99].

Maytansinoid is an antimitotic tubulin inhibitor derived from maytansine, whose mechanisms are similar to those of vinca alkaloids; however, its efficiency in killing tumor cells in vitro shows 100 to 1000 times more potent than existing anticancer drugs in clinical trials [100,101]. N^2′^-deacetyl-N^2′^-(3-mercapto-1-oxopropyl)-maytansine (DM1) and N^2′^-deacetyl-N^2′^-(4-mercapto-4-methyl-1-oxopentyl)-maytansine (DM4) are the most commonly synthesized payloads that applied in ADC pipeline [102]. The approved Trastuzumab emtansine (Kadcyla) adopted the DM1, while multiple ADCs in the clinical trials chose DM1 or DM4 as their toxins.

**Figure 16:**
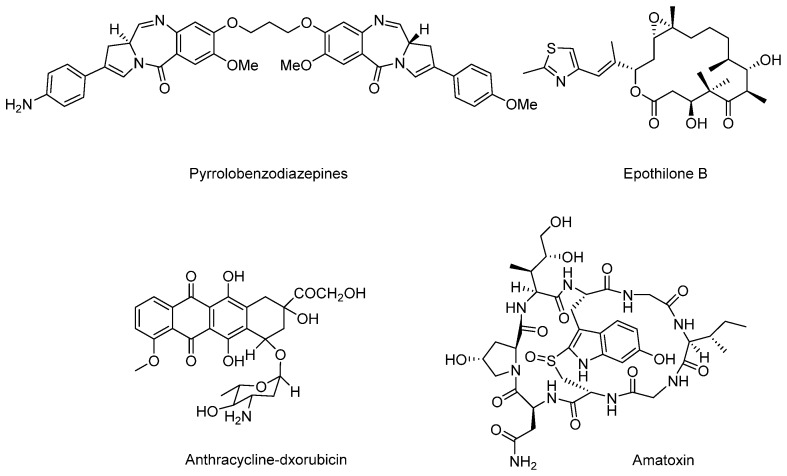
Structures of several terrestrial payloads.

Except for these four main payloads, there are also some other potential natural products that act as payloads, such as pyrrolobenzodiazepines (PBDs), anthracyclins, amatoxins, epothilones, and so on (Figure 16). PBDs are isolated from Streptomycin sp. and kill tumor cells by covalently binding to the minor groove of DNA. For ADCs in clinical trials, SGN-CD33A and SGN-CD70A adopt PBDs to connect with mAbs via protease-sensitive linkers and are in phase I/II clinical trials [103]. Anthracyclines are a kind of potent chemotherapeutics derived from *Streptomyces peucetius* var. caesius, which can be available for treatments of leukemia, lymphoma, breast cancer, ovarian cancer, and many other malignancies [104]. Milatuzumab doxorubicin takes doxorubicin as the payload and is in phase I/II clinical trials in patients with multiple myeloma (NCT01101594). Amatoxins are bicyclic octapeptides discovered from the poisonous mushroom *Amanita phalloides*, which is an efficient and selective inhibitor of RNA polymerase II to inhibit protein synthesis [105].

Recently, anti-PSMA-α-amanitin adopts amatoxins as the payload, conjugates to mAb via a protease-sensitive linker, and exerts favorable antitumor activity. However, details of the amanitin-based ADC have not been disclosed [106]. Compared with other toxins, amatoxins have great potential for their advantages of higher solubility, uniformity, and hydrophilicity. Epothilones are macrolides isolated from *Sprangium cellulosum*. Epothilones have a similar mechanism compared to that of paclitaxel, and are superior to paclitaxel in terms of antineoplastic activity, safety, solubility, and synthetic methods, which enable them to be the next generation of potentially antimitotic drugs [107,108].

#### 3.5.2. Marine Payloads

Marine organisms are regarded as an abundant natural product sources that produce a variety of marine toxins with antineoplastic activity; some toxins even reach nanomolar or picomolar cytotoxic activity [109,110].

**Figure 17:**
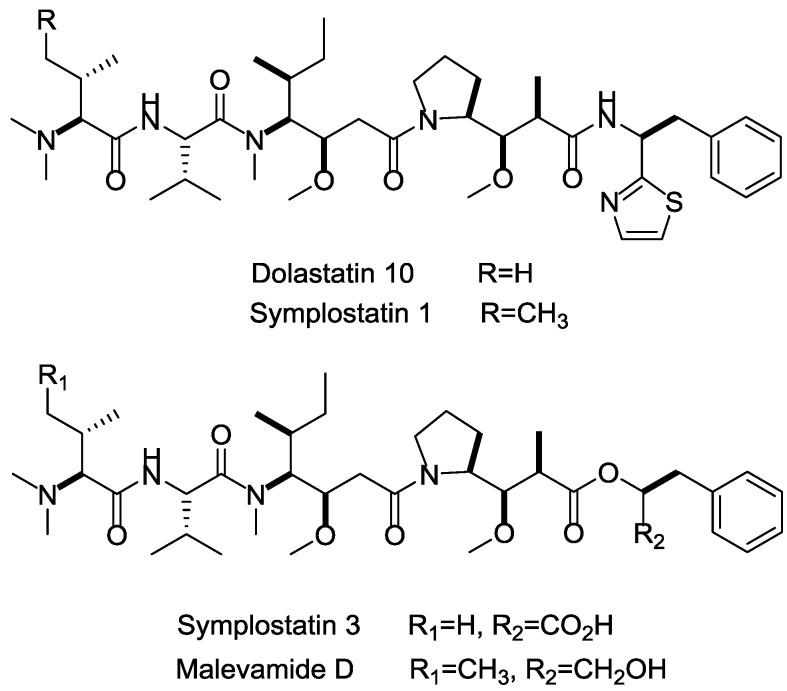
Structures of marine payloads.

Among the marine cytotoxins, auristatins and their derivatives (mainly MMAE and MMAF) take very important position in ADCs payloads. Auristatin is an efficient antimitotic toxin derived from the natural product dolastatin 10, which has an effective pentapeptide with unique amino acids discovered from the sea hare *Dolabella auricularia* [111]. Auristatins can interact with protein tubulins to inhibit microtubule formation and stop cell division during metaphase. Except for MMAE and MMAF modified from dolastatins 10 [83], plenty of other analogs, such as symplostatins 1, symplostatins 3, and malevamide D, also possess high tumor-killing activity (Figure 17) [112,113,114]. The brentuximab vedotin (Adcetris), developed by Seattle Genetics, adopted MMAE as the payload, and has been a success story with regard to marine drugs used in ADCs [115]. In current clinical trials, approximately half of these ADCs applied MMAE or MMAF as cytotoxinic drug (e.g., Glembatumumab vedotin, DCDS-4501A, AGS-16C3F, etc.).

#### 3.5.3. Marine Toxins

**Figure 18:**
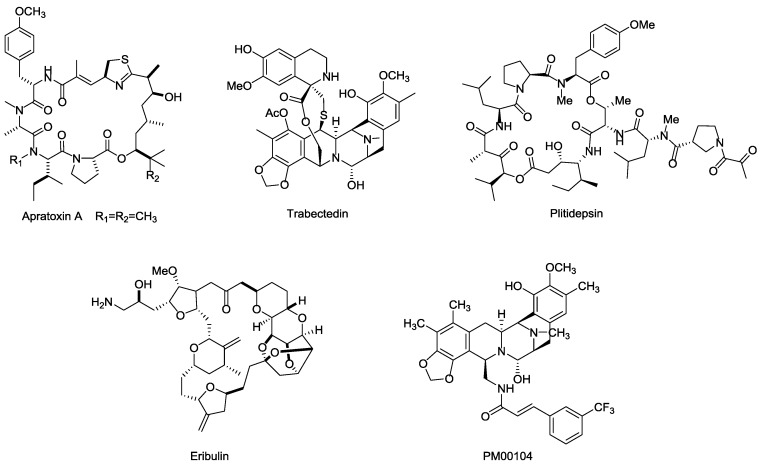
Structures of several marine toxins.

Besides the marine payloads for ADCs, more launched marine toxin drugs are also summarized in this section. Apratoxins are a type of cyclic depsipeptide with potent cytotoxinic activity (Figure 18). Apratoxin A, isolated from ocean cyanobacteria *Lyngbya* sp., exhibits highly potent antitumor effects via inhibiting cell division during the G1-phase and apoptosis, and displays excellent cytotoxicity in vitro with IC_50_ values ranging from 0.36 nM in LoVo cancer cells to 0.52 nM in KB cancer cells [116,117].

Both Trabectedin (ET-743, Yondelis^®^) and Plitidepsin (dehydrodidemnin B, Aplidin^®^) were highly potent anticancer drugs isolated from ascidian Ecteinascida turbinate in the Caribbean, which can kill tumor cells by binding to the minor groove of DNA and activating tumor cell apoptosis and multiple mechanisms [118,119,120,121]. Specifically, Trabectedin is a well-tolerated drug that lacks cumulative organ-specific toxicity and has completed phase II/III clinical trials for prostate and ovarian cancer and some other solid tumors (NCT00072670, NCT00113607, NCT00786838). Plitidepsin exhibits excellent cytotoxicity in vitro against DLBC and Burkitt's cells at 0.5 nM and 9 nM, respectively. The approved indication for Plitidepsin is for the treatment of multiple myeloma, while the research of Plitidepsin against lymphoma, RCC, and other solid tumors are still on the way [122,123,124].

Eribulin (E7389, Halaven^®^) is a highly potent analog of the famous marine natural product halichondrin B, isolated from the sponge *Halichondria okadai*. It exhibits a potential therapeutic effect against various cancers by microtubule inhibition [125]. Eribulin showed considerable anticancer activity in vitro, with IC_50_ values ranging from 0.06 nM to 0.3 nM in BT549 cancer cells [126,127], and was approved by FDA for the treatment of metastatic breast cancer in 2010. Further clinical trial studies focusing on other indications, such as lung, ovarian, and pancreatic cancers are ongoing [128].

PM00104 (Zalypsis^®^) is an analogs of (−)-Jorumycin, which isolated from a mollusk, *Jorunna funebris* [129], and exhibits potential anti-proliferative activity at nanomolar concentrations for the treatment of myelomas. The mechanism of PM00104 is similar to that of Trabectedin, including binding to DNA, leading DNA double-strand to breakages, cell cycle arresting in the S-phase, and cell apoptosis [130,131,132].

## 4. Conclusions and Future Directions

Research findings indicate that marine toxins have immense potential for the development of ADCs and simultaneously draw public attention to marine drug research [133,134]. In recent years, ADCs have developed rapidly since Adcetris and Kadcyla were successfully approved, while an increasing number of pharmaceutical companies have come into this research field. In the aspect of ADC design, with the rapid development of oncomolecularbiology, a multitude of specific tumor antigens (receptors, glycoproteins, etc.) are appraised and applied in medical diagnoses, which provides highly possibility for the targeting of anticancer drug development. The monoclonal antibody as the targeting part developed from the initial murine mAb to currently mature humanized mAb, and even fully human mAb, which immensely ameliorates the immunogenicity problem. In order to implement the transition from hematologic tumors to solid tumors, antibody miniaturization will be a new strategy for developing ADCs. The linker has made remarkable headway, and is now in a comparatively mature stage, while more innovative linkers are being created with modern chemical biology conceptions. Presently, common payloads in the pipeline mainly focus on auristatins and maytansines; however, a series of studies have suggested that more marine toxins in addition to auristatins have the potential to be effective ADC payloads.

The inhomogeneity problem is still the major bottleneck in current ADC development, which negatively affects both pharmaceutical production and quality control. Nonetheless, site-specific conjugation technology has caught numerous pharmaceuticals companies’ eyes for solving the inhomogeneity problem to some extent, pointing out the way to further development of ADCs. With the advancement of conjugation technology, a variety of conjugated groups have been designed and connect with many different drugs by the diversified modification of linkers. Multivalent-coupling ADCs will be developed to improve efficacy via connecting several synergistic small molecules in one antibody. ThioBridge™, GlycoConnect™, and many other site-specific conjugation technologies will become a prevalent trend and accelerate ADC development into a brand new era.

## Figures and Tables

**Table 1:** ADCs in clinical research.

ADCs	Phase	Target	Antibody	Linker	Payload	Indication(s)
Inotuzumab ozogamicin	Phase III	CD22	Hz IgG_4_	Hydrazone	Calicheamicin	NHL, ALL, CML
Gemtuzumab ozogamicin	Phase II	CD33	Hz IgG_4_	Hydrazone	Calicheamicin	AML, APL
MDX-1203	Phase I	CD70	n.d.	Valine-citrulline	Duocarmycin	RCC, NHL
Glembatumomab vedotin	Phase II	GPNMB	Hu IgG_2_	Valine-citrulline	MMAE	Breast cancer, melanoma
Anti-PSMA ADC	Phase I/II	PSMA	Hu IgG_1_	Valine-citrulline	MMAE	Prostatic cancer
RG-7593/DCDT-2980S	Phase I/II	CD22	Hz IgG_1_	Valine-citrulline	MMAE	NHL, DLBCL
RG-7596/DCDS-4501A	Phase I/II	CD79b	Hz IgG_1_	Valine-citrulline	MMAE	NHL, DLBCL
RG7599/DNIB-0600A	Phase I/II	NaPi2b	Hz IgG_1_	n.d.	MMAE	Ovarian cancer, NSCLC
MLN0264	Phase I/II	GCC	n.d.	n.d.	MMAE	Pancreatic cancer, gastric carcinoma
ASG-22M6E	Phase I	Nectin-4	Hu IgG_1_	Valine-citrulline	MMAE	Solid tumors
ASG-5ME	Phase I	SLC44A4	Hu IgG_2_	Valine-citrulline	MMAE	Pancreatic cancer, prostatic cancer
AGS-67E	Phase I	CD37	Hu IgG_2_	Valine-citrulline	MMAE	Lymphoma, AML
BAY-79-4620	Phase I	CA-IX	Hu IgG_1_	Valine-citrulline	MMAE	Solid tumors
RG7458/DMUC-5754A	Phase I	MUC16	IgG_1_	n.d.	MMAE	Ovarian cancer, pancreatic cancer
RG7636	Phase I	ETBR	n.d.	n.d.	MMAE	Melanoma
AGS-15ME	Phase I	SLITRK6	Hu IgG_2_	Cleavable linker	MMAE	Urothelial neoplasms
HuMax^®^-TF	Phase I	TF	Hu IgG_1_	Valine-citrulline	MMAE	Solid tumors
SGN-LIV1A	Phase I	LIV-1	Hz IgG_1_	Valine-citrulline	MMAE	Breast cancer
AGS-16C3F	Phase I/II	ENPP3	Hu IgG_2_	MC	MMAF	RCC
SGN-CD19A	Phase I/II	CD19	Hz IgG_1_	MC	MMAF	Lymphoma, DLBCL
ABT-414	Phase I/II	EGFR	Hu IgG_1_	n.d.	MMAF	Solid tumors, glioma, SCC
GSK-2857916	Phase I	BCMA	Hz IgG_1_	MC	MMAF	MM
AGS-16M8F	Phase I	ENPP3	Hu IgG_2_	MC	MMAF	RCC
A1-mc-MMAF	Phase I	5T4	Hz IgG_1_	MC	MMAF	Solid tumors
IMGN-529	Phase I/II	CD37	IgG_1_	Thioether	DM1	NHL, CLL, DLBLC
Lorvotuzumab mertansine	Phase I/II	CD56	Hz IgG_1_	SPP	DM1	SCLC, MM
AMG-172	Phase I	CD27L	Hu IgG_1_	MCC	DM1	ccRCC
IMGN-289	Phase I	EGFR	Hz IgG	SMCC	DM1	Solid tumors
AMG-595	Phase I	EGFRvIII	n.d.	SMCC	DM1	Glioma
SAR-3419	Phase II	CD19	Hz IgG_1_	SPDB	DM4	NHL, DLBCL
BT-062	Phase II	CD138	Ch IgG_4_	SPDB	DM4	MM
BAY-94-9343	Phase I/II	Mesotherin	Hu IgG_1_	SPDB	DM4	Solid tumors
IMGN-853	Phase I/II	FOLR1	IgG_1_	n.d.	DM4	Solid tumors
SAR-566658	Phase I	CA6	Hu IgG_1_	SPDB	DM4	Solid tumors
IMGN-388	Phase I	Integrinαvβ3	Hu IgG_1_	SPDB	DM4	Solid tumors
BIIB-015	Phase I	Cripto	Hz IgG_1_	SPDB	DM4	Solid tumors
Labetuzumab-SN-38	Phase I/II	CD66e	Hz IgG_1_	Phenylalanine-lysine	SN38	CRC
IMMU-132	Phase I/II	TROP-2	Hz IgG_1_	CL2A	SN38	Epithelial carcinomas, solid tumors
SGN-CD33A	Phase I/II	CD33	Hz IgG_1_	Valine-alanine	PBDs	AML, APL
SGN-CD70A	Phase I	CD70	n.d.	Valine-alanine	PBDs	RCC, lymphoma
SC16LD6.5	Phase I/II	Fyn3	SC16	n.d.	D6.5	SCLC
Milatuzumab doxorubicin	Phase I/II	CD74	Hz IgG_1_	Hydrazone	Doxorubicins	MM
SYD985	Phase I	HER2	Hz IgG	n.d.	n.d.	Solid tumors
IGN523	Phase I	CD98	Hz IgG	n.d.	n.d.	AML

n.d.: not disclosed.

**Table 2:** Carcinomas and Specific Antigens.

Indications	Antigen Targets
NHL	CD19, CD20, CD21, CD22, CD37, CD70, CD72, CD79a/b, CD180
HL	CD30
AML	CD33, CD98
MM	CD56, CD74, CD138, ETBR
Lung cancer	CD24, CD56, CD326, Cripto, FAP, Mesothelin, GD2, 5T4, NaPi2b, FOLR1, Integrinαvβ3, Fyn3
CRC	CD74, CD174, CD227, CD326, CRIPTO, FAP, ED-B, CD66e
Pancreatic cancer	CD74, CD227, nectin-4, CA19-9, MUC-4, MUC16, alpha v beta6, GCC
Breast cancer	CD174, GPNMB, CRIPTO, nectin-4, LIV1A
Ovarian cancer	MUC16, TIM-1, Mesothelin, NaPi2b
Melanoma	GD2, GPNMB, ED-B, PMEL 17, ETBR
Prostate cancer	PSMA, STEAP-1, TENB2
Renal cancer	CAIX, TIM-1, CD27L, CD70, ENPP3
Mesothelioma	Mesothelin
Urothelial cancer	SLITRK6
Glioma	EGFRvIII

**Table 3:** FDA-Approved Therapeutic Antibodies for Cancer.

Generic Name	Trade Name	Target	Antibody	Indication(s)	Year of First Approval
Edrecolomab	Panorex	EpCAM	Murine IgG_2_	CRC	1995
Ibritumomab tiuxetan	Zevalin	CD23	Murine IgG_1_	NHL	2002
^131^I-labeled tositumumab	Bexxar	CD20	Murine IgG_2_	NHL	2003
Rituximab	Rituxan	CD20	Chimeric IgG_1_	B-NHL	1997
^131^I-labeled ch-TNT	n.d.	n.d.	Chimeric IgG_1_	Lung cancer	2003
Cetuximab	Erbitux	EGFR	Chimeric IgG_1_	CRC	2004
Brentuximab	Adcetris	CD30	Chimeric IgG_1_	ALCL, HL	2011
Trastuzumab	Herceptin	HER2	Humanized IgG_1_	Breast cancer	1998
Gentuzumab ozogamcin	Mylotarg	CD33	Humanized IgG_4_	Leukemia	2000
Alemtuzumab	Campath	CD52	Humanized IgG_1_	B-CLL	2001
Bevacizumab	Avastin	VEGF	Humanized IgG_1_	CRC, lung, breast cancer	2004
Nimotuzumab	TheraCIM	EGFR	Humanized IgG_1_	Epithelial cancer	2005
Pertuzumab	Perjeta	HER2	Humanized IgG_1_	Breast cancer	2012
Panitumumab	Vectibix	EGFR	Human IgG_2_	CRC	2007
Ofatumumab	Arzerra	CD20	Human IgG_1_	CLL	2009
Ipilimumab	Yervoy	CTLA4	Human IgG_1_	Melanoma	2011

n.d.: not disclosed.

**Table 4:** Representative Triggers in Linkers.

Cleavable Linker	Non-Cleavable Linker
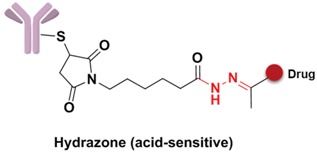	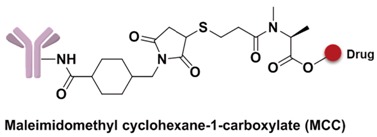
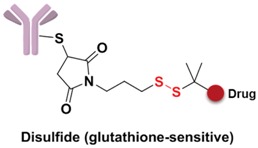	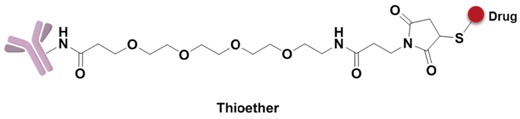
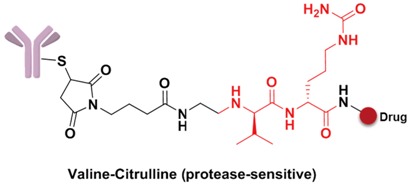	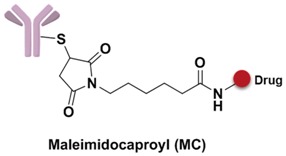

## Abbreviations

AcBut	4-(4-acetylphenoxy)butanoic acid
ADAs	allergic reactions and anti-drug antibodies
ADC	antibody–drug conjugate
ADCC	antibody-dependent cell-mediated cytotoxicity
ALCL	anaplastic large cell lymphoma
ALL	acute lymphoblastic leukemia
AML	acute myelocytic leukemia
APL	acute promyelocytic leukemia
BCMA	B cell maturation antigen
ccRCC	clear cell renal cell carcinoma
CDC	complement dependent cytotoxicity
Ch	chimeric
CLL	chronic lymphocytic leukemia
CML	chronic myeloid leukemia
CRC	colorectal carcinoma
CRPC	castration resistant prostate cancer
CTLA4	cytotoxic T-lymphocyte antigen 4
DARs	Drug: antibody ratios
DLBCL	diffuse large B cell lymphoma
DM1	N^2′^-deacetyl-N^2′^-(3-mercapto-1-oxopropyl)-maytansine
DM4	N^2′^-deacetyl-N^2′^-(4-mercapto-4-methyl-1-oxopentyl)-maytansine
DTM	dithiopyridylmaleimide
EGFR	epidermal growth factor receptor
EpCAM	epithelial cell adhesion molecule
ETBR	endothelin B receptor
Fab	antigen-binding fragment
Fc	crystalline fragment
FcRn	human neonatal Fc receptor
FDA	Food and Drug Administration
FGE	formylglycine-generating enzyme
GCC	Guanylyl cyclase C
GPNMB	glycoprotein nonmetastatic melanoma protein b
HER2	human epidermalgrowth factor receptor-2
HL	Hodgkin’s lymphoma
Hu	human
Hz	humanized
mAb	monoclonal antibody
MC	maleimidocaproyl
MCC	maleimidomethyl cyclohexane-1-carboxylate
MM	multiple myeloma
MMAE	monomethyl auristatin E
MMAF	monomethyl auristatin F
NHL	non-hodgkin’s lymphoma
NSCLC	non-small-cell lung cancer
PAB	para-aminobenzyl
pAcPhe	*p*-acetylphenylalanine
PBDs	pyrrolobenzodiazepines
PEG	polyethylene glycol
PK	pharmacokinetic
PSMA	prostate specific membrane antigen
RCC	renal cell carcinoma
SCC	squamous cell carcinoma
scFv	single-chain variable fragment
SCLC	small-cell lung cancer
TF	tissue factor
TPBG	trophoblast glycoprotein
VEGF	vascular endothelial growth factor
